# Antibiotic Resistance in the Elderly: Mechanisms, Risk Factors, and Solutions

**DOI:** 10.3390/microorganisms12101978

**Published:** 2024-09-30

**Authors:** Nikolaos Theodorakis, Georgios Feretzakis, Christos Hitas, Magdalini Kreouzi, Sofia Kalantzi, Aikaterini Spyridaki, Iris Zoe Boufeas, Aikaterini Sakagianni, Evgenia Paxinou, Vassilios S. Verykios, Maria Nikolaou

**Affiliations:** 1 Department of Cardiology, 65+ Clinic, Amalia Fleming General Hospital, 14, 25th Martiou Str., 15127 Melissia, Greece; n.theodorakis@flemig-hospital.gr (N.T.); ch.chitas@flemig-hospital.gr (C.H.); m.nikolaou@flemig-hospital.gr (M.N.); 2 School of Medicine, National and Kapodistrian University of Athens, 75 Mikras Asias, 11527 Athens, Greece; 3 School of Science and Technology, Hellenic Open University, 18 Aristotelous Str., 26335 Patras, Greece; georgios.feretzakis@ac.eap.gr (G.F.); paxinou.evgenia@ac.eap.gr (E.P.); 4 Department of Internal Medicine, 65+ Clinic, Amalia Fleming General Hospital, 14, 25th Martiou Str., 15127 Melissia, Greece; kreouzi.m@live.unic.ac.cy (M.K.); sofia_kalanji@yahoo.gr (S.K.); kspyridaki@yahoo.gr (A.S.); 5 Barts and The London School of Medicine and Dentistry, Queen Mary University of London, 64 Turner Street, London E1 2AD, UK; i.z.boufeas@smd22.qmul.ac.uk; 6 Intensive Care Unit, Sismanogelio General Hospital, 37 Sismanogleiou Str., 15126 Marousi, Greece; sakagianni@sismanoglio.gr

**Keywords:** antibiotic resistance, immunosenescence, multidrug-resistant organisms, infectious diseases, antimicrobials, vaccinations, antimicrobial stewardship, machine learning, aging, elderly

## Abstract

Antibiotic resistance presents a critical challenge in healthcare, particularly among the elderly, where multidrug-resistant organisms (MDROs) contribute to increased morbidity, mortality, and healthcare costs. This review focuses on the mechanisms underlying resistance in key bacterial pathogens and highlights how aging-related factors like immunosenescence, frailty, and multimorbidity increase the burden of infections from MDROs in this population. Novel strategies to mitigate resistance include the development of next-generation antibiotics like teixobactin and cefiderocol, innovative therapies such as bacteriophage therapy and antivirulence treatments, and the implementation of antimicrobial stewardship programs to optimize antibiotic use. Furthermore, advanced molecular diagnostic techniques, including nucleic acid amplification tests and next-generation sequencing, allow for faster and more precise identification of resistant pathogens. Vaccine development, particularly through innovative approaches like multi-epitope vaccines and nanoparticle-based platforms, holds promise in preventing MDRO infections among the elderly. The role of machine learning (ML) in predicting resistance patterns and aiding in vaccine and antibiotic development is also explored, offering promising solutions for personalized treatment and prevention strategies in the elderly. By integrating cutting-edge diagnostics, therapeutic innovations, and ML-based approaches, this review underscores the importance of multidisciplinary efforts to address the global challenge of antibiotic resistance in aging populations.

## 1. Introduction

Population aging is a global phenomenon that affects most developed societies [1]. This leads to a significant increase in the healthcare burden of the elderly population. Geriatric patients are susceptible to infections, particularly by multidrug-resistant organisms (MDROs). This results in an increased prevalence of serious and difficult-to-treat infections, leading to significant impacts on morbidity, mortality, length of hospital stays and health costs [2]. In Greece during 2020 the mortality rate due to infections was 40.1 per 100,000 in patients aged 65–79 years, while in patients aged 80+, it was five times higher (389.8 per 100,000) [3].

Antibiotic resistance is primarily a result of the overuse or misuse of antimicrobials, which leads to the selective growth of resistant strains [4]. The elderly are disproportionately affected by MDROs due to a constellation of factors. Patient-specific factors include immunosenescence, frailty, sarcopenia, cognitive dysfunction, multimorbidity, and polypharmacy [5]. Furthermore, frequent exposure to hospital care facilities or institutionalization leads to increased contact with MDROs. These environments, combined with invasive medical interventions such as the use of urinary catheters and central lines, facilitate the colonization by MDROs, which can cause severe infections [6].

This review aims to analyze the mechanisms of antibiotic resistance in the elderly as well as the factors that make the elderly especially susceptible to infections from MDROs. Additionally, we analyze strategies to mitigate the burden of such infections in the elderly. Finally, we provide a brief overview of the role of machine learning (ML) in improving diagnosis, treatment, and prevention of antibiotic resistance in elderly populations.

## 2. Key MDROs in the Elderly

The prevalence and distribution of MDROs can vary significantly between countries, hospitals, and even between departments within the same hospital. Additionally, the frequency of MDROs differs among various types of infections and between patients with differing comorbidities and levels of immune function. These variations highlight the importance of tailoring treatment strategies to local epidemiological data, as this provides the most accurate and relevant information on resistance patterns within a specific healthcare setting. Despite this variability, published studies offer valuable insights into the most frequently encountered MDROs in elderly patients, particularly in the context of the most common infections in this population: urinary tract infections (UTI) and pneumonia. These overviews can serve as a useful guide for clinicians, especially in situations where local data are incomplete or unavailable.

For UTI, the most prevalent MDROs include *Enterobacteriaceae* (such as *Escherichia coli*, *Klebsiella pneumoniae*, *Proteus mirabilis*, and *Enterobacter cloacae*), *Pseudomonas aeruginosa*, *Enterococcus faecalis* and *Enterococcus faecium* (including vancomycin-resistant enterococci, VRE), and *Acinetobacter baumannii* [7]. In pneumonia cases, the most commonly implicated MDROs are *Streptococcus pneumoniae*, methicillin-resistant *Staphylococcus aureus* (MRSA), *Enterobacteriaceae* (*E. coli* and *K. pneumoniae*), *P. aeruginosa*, and *A. baumannii* [8,9].

**Figure 1:**
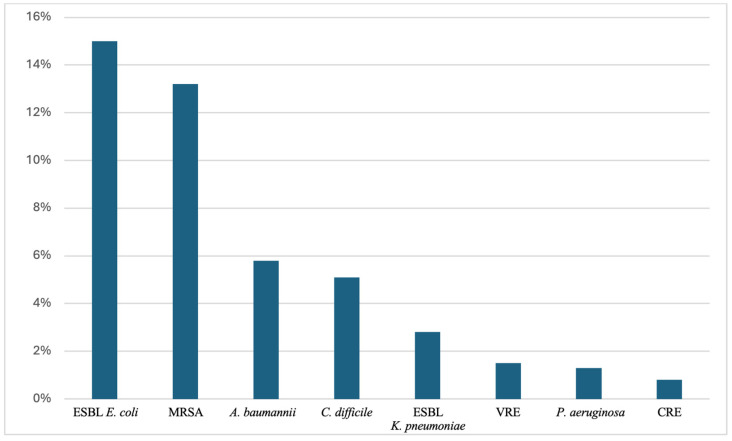
Median Prevalence of MDROs Among Elderly Residents in LTCFs. Abbreviations. CRE (carbapenem-resistant *Enterobacteriaceae*); ESBL (extended-spectrum β-lactamase); LTCFs (long-term care facilities); MDROs (multidrug-resistant organisms); MRSA (methicillin-resistant *Staphylococcus aureus*); VRE (vancomycin-resistant *Enterococcus*).

The median prevalence of the most common MDROs in the elderly can be concluded from a recent study with data from long-term care facilities worldwide, as illustrated in Figure 1. This study revealed that extended-spectrum beta-lactamase (ESBL)-producing *Enterobacteriaceae* have the highest median prevalence at 11.6%, with *E. coli* contributing 15.0% and *K. pneumoniae* 2.9%. MRSA follows closely with a median prevalence of 13.2%, while *Clostridioides difficile* accounts for 5.1%. Multidrug-resistant (MDR) *A. baumannii* stands at 5.8%, and VRE is has a median prevalence of 1.5%. Carbapenem-resistant *Enterobacteriaceae* (CRE) has a median prevalence of 0.8%, while MDR *P. aeruginosa* stands at 1.3% [10].

### 2.1. MRSA

MRSA is one of the most notorious antibiotic-resistant pathogens, particularly in hospital and long-term care settings, where it causes severe skin, respiratory, and bloodstream infections. The primary mechanism of resistance in MRSA involves the acquisition of the *mecA* gene, which encodes an altered penicillin-binding protein (PBP2a) [11]. PBP2a has a reduced affinity for beta-lactam antibiotics, including methicillin, rendering these drugs ineffective. Additionally, MRSA employs efflux pumps like NorA, which actively expel antibiotics such as fluoroquinolones from the bacterial cell, reducing their intracellular concentration and efficacy [12].

MRSA is also known for its ability to form biofilms, particularly on indwelling medical devices like catheters and prosthetic joints. Biofilms are structured communities of bacteria encased in a self-produced extracellular matrix, which shields the bacteria from antibiotics and immune defenses, allowing them to persist and cause chronic infections [11]. The horizontal gene transfer of resistance determinants through plasmids and transposons further contributes to the spread of resistance among different strains of *S. aureus* [13].

### 2.2. VRE

*E. faecium* and *E. faecalis* are the primary species responsible for VRE infections, which are particularly challenging to treat in elderly patients with weakened immune systems. Resistance to vancomycin in VRE is primarily mediated by the *vanA* or *vanB* gene clusters, which modify the terminal D-Ala-D-Ala dipeptides in the bacterial cell wall to D-Ala-D-Lac, reducing vancomycin’s binding affinity. In addition to vancomycin resistance, VRE can exhibit resistance to other antibiotics through beta-lactamase production and target site alterations, complicating treatment regimens [14].

Like MRSA, VRE can form biofilms, particularly in the gastrointestinal tract, which protects the bacteria from antibiotics and host defenses [15]. Horizontal gene transfer plays a significant role in the spread of vancomycin-resistance genes between enterococcal species, further amplifying the problem in healthcare settings [16].

### 2.3. S. pneumoniae

*S. pneumoniae* is a leading cause of pneumonia, meningitis, and sepsis in elderly populations. The development of resistance in *S. pneumoniae* is largely attributed to alterations in penicillin-binding proteins (PBPs). These alterations occur due to the acquisition of mosaic genes that encode PBPs with reduced binding affinity for beta-lactam antibiotics, resulting to ineffectiveness of beta lactams [17].

In addition to beta-lactam resistance, MDR *S. pneumoniae* can resist macrolides through the *ermB* gene, which encodes ribosomal methylases that modify the macrolide binding site, and the *mefA* gene, which encodes efflux pumps that actively expel macrolide antibiotics from the bacterial cell [18]. The formation of biofilms is another strategy employed by *S. pneumoniae* to resist antibiotics, particularly in cases of chronic respiratory infections [19].

### 2.4. Enterobacteriaceae

ESBL-producing *Enterobacteriaceae* and CRE are a major threat in healthcare settings. ESBL-producing *Enterobacteriaceae* produce beta-lactamases that hydrolyze all penicillin and cephalosporin antibiotics and are resistant to penicillinase inhibitors such as sulbactam and tazobactam [20]. *K. pneumoniae* carbapenemase (KPC) have the ability to resist nearly all available beta-lactam antibiotics, including carbapenems, which are often considered last-resort treatments. CRE bacteria, such as *K. pneumoniae* and *E. coli*, produce carbapenemase enzymes that hydrolyze the beta-lactam ring of carbapenem antibiotics, rendering them ineffective. The most common carbapenemases include KPC, New Delhi metallo-beta-lactamase (NDM), and oxacillinase (OXA)-48 [20].

In addition to carbapenemase production, CRE bacteria employ porin mutations that reduce the uptake of antibiotics, particularly carbapenems. The loss or alteration of OmpK36 in *K. pneumoniae* is associated with high levels of carbapenem resistance [21]. CRE also use efflux pumps, such as AcrAB-TolC, to expel multiple classes of antibiotics, further complicating treatment [22]. The transfer of resistance genes through plasmids and transposons contributes to the rapid dissemination of carbapenem resistance among *Enterobacteriaceae* [23].

### 2.5. C. difficile

*C. difficile* is a major cause of healthcare-associated diarrhea and colitis, particularly in elderly patients who have undergone prolonged antibiotic therapy. Antibiotic resistance in *C. difficile* is primarily associated with fluoroquinolone resistance, due to mutations in the *gyrA* and *gyrB* genes, which encode DNA gyrase, the target of fluoroquinolones. Resistance to other antibiotics, such as metronidazole and vancomycin, has also been reported, though the clinical relevance of these resistant strains is still under investigation [24].

*C. difficile’s* ability to form spores enhances its persistence in the healthcare environment, as spores are resistant to disinfectants, heat, and other environmental stressors. This spore-forming capability complicates infection-control measures and contributes to the recurrence of *C. difficile* infections (CDI) [25].

### 2.6. Mycobacterium tuberculosis

Tuberculosis (TB) remains a significant global health threat, and elderly individuals, particularly those with weakened immune systems, are at increased risk of developing drug-resistant TB. MDR-TB is defined as resistance to at least isoniazid and rifampin, while extensively drug-resistant (XDR) TB refers to additional resistance to fluoroquinolones and second-line injectable drugs [26].

Resistance in *M. tuberculosis* primarily arises from mutations in drug target genes. For example, mutations in *katG* (encoding catalase-peroxidase) result in resistance to isoniazid, while mutations in *rpoB* (the target of rifampin) confer resistance to rifampin. Additionally, *M. tuberculosis* employs efflux pumps and cell wall modifications that limit the penetration of antibiotics, further complicating treatment [27].

### 2.7. P. aeruginosa

*P. aeruginosa* is a highly versatile pathogen that causes severe infections in immunocompromised elderly individuals, particularly those with chronic respiratory diseases such as chronic obstructive pulmonary disease (COPD). *P. aeruginosa* is intrinsically resistant to many antibiotics due to its low outer membrane permeability, the presence of multiple efflux pumps (such as MexAB-OprM and MexXY), mutations in porins, and the production of beta-lactamases, including metallo-beta-lactamases (MBLs) [28]. Additionally, *P. aeruginosa* can form robust biofilms, particularly in the lungs of patients with cystic fibrosis or ventilator-associated pneumonia, which further protects the bacteria from antibiotics and the host immune response [28,29]. Horizontal gene transfer of resistance elements through plasmids and integrons also contributes to the rapid spread of multidrug resistance in this pathogen [30].

### 2.8. A. baumannii

*A. baumannii* is a notorious pathogen in intensive care units, where it causes ventilator-associated pneumonia, bloodstream infections, and wound infections in elderly patients. *A. baumannii* exhibits resistance to a wide range of antibiotics, including beta-lactams, aminoglycosides, and fluoroquinolones, through a combination of efflux pumps, beta-lactamase production, target site mutations, and porin modifications [31].

One of the most concerning resistance mechanisms in *A. baumannii* is the production of OXA-type carbapenemases, which confer resistance to carbapenems, making infections difficult to treat [32]. The bacterium also forms biofilms on medical devices and wounds, further complicating eradication efforts [33]. Horizontal gene transfer plays a critical role in the dissemination of resistance genes among *A. baumannii* species, particularly in healthcare environments [34].

The mechanisms of the most prevalent MDROs and their most common infections in the elderly are summarized in Table 1.

## 3. Risk Factors for Infections from MDROs in the Elderly

### 3.1. Immunosenescence

Immunosenescence refers to the gradual decline in immune function that occurs with aging, affecting both the innate and adaptive branches of the immune system. Innate immunity is compromised by a reduction in the phagocytic activity of neutrophils and macrophages as well as impaired production of pro-inflammatory cytokines such as IL-1, IL-6, and TNF-α, which are critical for mounting an early response to infection. The function of Toll-like receptors (TLR), which recognize pathogen-associated molecular patterns and initiate immune responses, is also impaired in the elderly, reducing their ability to detect and respond to infections [35].

On the adaptive immunity side, aging leads to thymic involution, which significantly reduces the production of naïve T cells. The limited T-cell receptor diversity in elderly individuals hampers their ability to recognize and respond to new pathogens, making them more susceptible to infections caused by emerging or resistant bacteria. The B-cell compartment is similarly affected, with aging leading to reduced antibody production and affinity, leaving the elderly vulnerable to recurrent infections and less responsive to vaccination. As a result, elderly individuals have a diminished capacity to clear infections, which contributes to the persistence and spread of antibiotic-resistant pathogens in healthcare settings [36].

### 3.2. Polypharmacy

Polypharmacy, defined as the concurrent use of five or more medications, is a common phenomenon among elderly individuals, particularly those with multiple chronic conditions. Polypharmacy increases the risk of adverse drug effects and interactions and can complicate the use of antibiotics. Specifically, proton pump inhibitors can increase susceptibility to infections from resistant enteropathogenic bacteria, including *C. difficile* [37]. Furthermore, medications such as proton pump inhibitors, non-steroidal anti-inflammatories, corticosteroids, and metformin can cause dysbiosis, which can lead to the overgrowth of resistant organisms, including *C. difficile* and ESBL-producing *Enterobacteriaceae* [38]. Additionally, polypharmacy increases the likelihood of inappropriate antibiotic use, particularly when antibiotics are received without a prescription or prescribed for nonspecific symptoms, such as confusion or lethargy, which can be a result of adverse effect of medications [39].

### 3.3. Sarcopenia and Malnutrition

Sarcopenia, defined as the progressive loss of skeletal muscle mass and function, is closely linked to frailty and increased susceptibility to infections, including those from MDROs. Sarcopenia is multifactorial and is particularly associated with age-related chronic low-grade inflammation (inflammaging) and functional hypogonadism in males [40]. Sarcopenia reduces metabolic reserves, impairs wound healing, and has been associated with immunosenescence [41]. Malnutrition, which often coexists with sarcopenia, further exacerbates immunosuppression by reducing the availability of essential nutrients required for immune cell function, affecting both innate and adaptive immunity. Deficiencies in key micronutrients, such as zinc, vitamin D, vitamin C, and selenium, are particularly detrimental, as they play critical roles in immune cell signaling, antioxidant defense, and pathogen clearance. For example, zinc is essential for all highly proliferative immune cells, while vitamin D modulates both innate and adaptive immune responses. The deficiency of these nutrients in the elderly patients increases their susceptibility to infections caused by *S. pneumoniae, E. coli*, and other resistant organisms [42].

### 3.4. Frailty and Decreased Mobility

Frailty is a syndrome characterized by decreased physiological reserve and resistance to stressors, leaving elderly individuals more vulnerable to infections and slower to recover from illness [43]. Frailty is associated with increased inflammation, as evidenced by elevated levels of C-reactive protein, IL-6, and TNF-α, which contribute to chronic immune activation and tissue damage. This chronic inflammatory state, often referred to as inflammaging, is linked to an increased risk of infections, particularly those caused by resistant organisms [44].

Frailty often coexists with decreased mobility, which limits an individual’s ability to recover from illness and increases their dependence on healthcare services. Prolonged immobility can lead to the development of pressure ulcers and chronic wounds, which serve as entry points for MRSA, VRE, and other resistant pathogens [45]. Immobility also increases the risk of hospital-acquired pneumonia (HAP), aspiration pneumonia, and UTI, both of which are common in frail elderly patients and are often caused by MDROs. In addition, decreased mobility is associated with prolonged hospital stays and increased use of invasive medical devices, further compounding the risk of resistant infections [46,47].

### 3.5. Cognitive Impairment

Elderly patients with dementia, such as Alzheimer’s disease, vascular dementia, or other neurodegenerative disorders, have an increased risk for infections. Cognitive dysfunction often leads to poor personal hygiene, frequent use of urinary catheters and feeding tubes, and difficulties adhering to infection prevention protocols, all of which increase the risk of bacterial transmission. Coupled with the fact that these patients frequently contact healthcare environments or reside in nursing homes, they are at particular risk for colonization and subsequent infections from MDROs. Furthermore, the fact that these patients are unable to retain independence in activities of daily living increases their contact with nurses and carers, which is often the factor that leads to transmission of MDROs, especially in hospital settings and long-term care facilities [48].

Another important factor is that infections in elderly patients with cognitive impairment often have an atypical presentation with the absence of typical symptoms such as fever or dysuria. Non-specific symptoms such as confusion, falls, or weakness might have a non-bacterial cause (e.g., dehydration or medication side effects), but in several cases, they might prompt the improper initiation use of broad-spectrum antibiotics, which promotes the development of MDROs [49].

### 3.6. Multimorbidity

Multimorbidity significantly increases the risk of infections from MDROs in elderly populations, as chronic diseases often impair the immune response and increase the likelihood of healthcare exposure. Comorbidities such as diabetes mellitus, chronic kidney disease (CKD), heart failure (HF), and COPD, compromise the immune system and lead to hospitalizations and frequent need for prolonged courses of broad-spectrum antibiotics, contributing to the colonization from MDROs [50,51,52,53].

Diabetes mellitus, for example, impairs the innate immunity through reduction of neutrophil chemotaxis, phagocytosis, and bactericidal activity. Diabetic patients are at an especially increased risk of skin and soft tissue infections caused by MRSA, ESBL-producing organisms, and *P. aeruginosa* [50]. Patients with CKD are particularly vulnerable to infections due to uremia-induced immune dysfunction and frequent use of dialysis catheters, which serve as entry points for *Staphylococcus epidermidis* (including MRSE), VRE, and CRE [51]. Patients with HF and COPD face an elevated risk of infections, particularly pneumonia. However, antibiotic misuse is alarmingly prevalent in the management of these conditions. A common issue arises when patients with cardiogenic pulmonary edema are given broad-spectrum antibiotics despite the absence of a confirmed infection, often based solely on slight elevations in C-reactive protein, which confers risk of colonization with MDRO. In the context of COPD, antibiotics should be reserved exclusively for infective exacerbations, as confirmed by the Antonissen criteria, to prevent unnecessary exposure and reduce antibiotic resistance risk [52,53].

Finally, immunosuppressive therapies, such as corticosteroids and biologic agents used to manage chronic inflammatory and autoimmune diseases, increase susceptibility to opportunistic infections caused by resistant organisms, including MDR/XDR TB [54].

### 3.7. Frequent Hospitalizations and Long-Term Care Facility Residency

Elderly individuals are frequently hospitalized or reside in long-term care facilities, both of which are environments with a high prevalence of MDROs. In long-term care facilities, elderly patients are often in close contact with one another, and the use of communal spaces and shared healthcare equipment facilitates the transmission of resistant bacteria. In hospitals, elderly patients are more likely to undergo invasive medical procedures, such as urinary catheterization, central venous catheterization, and mechanical ventilation, all of which increase the risk of colonization and infection by MDROs. As a result, when these patients present with signs or symptoms of an infection, they are often administered broad spectrum antibiotics such as piperacillin-tazobactam and meropenem to cover for the possibility of Gram-negative MDROs. This increases the risk of the development of more resistant organisms by selective pressure that promotes the survival of resistant strains, such as ESBL-producing *E. coli* and *K. pneumoniae*, creating a vicious cycle of resistance [55].

The misuse or prolonged use of antibiotics in elderly populations also contributes to the development of CDI, which are often associated with antibiotic-induced disruption of the gut microbiome. Recurrent CDI a major concern in elderly patients, as it increases the risk of colonization by resistant bacteria and contributes to the overall burden of antibiotic resistance in healthcare settings [56].

A summary of the risk factors for MDRO-related infections in the elderly is presented in Table 2.

## 4. Strategies to Combat Antibiotic Resistance in the Elderly

### 4.1. Novel Antibiotics

As the effectiveness of traditional antibiotics diminishes due to increasing resistance, the development of new antibiotics with novel mechanisms of action has become crucial. As previously discussed, the elderly population is disproportionately impacted by infections caused by MDROs, often facing more severe clinical outcomes. As a result, the novel antibiotics discussed here are particularly significant in the management MDRO-related infections in geriatric patients, offering chances for a better prognosis with decreased mortality.

Teixobactin represents a significant breakthrough in antibiotic development, discovered through the iChip technology that allows cultivation of bacteria in their natural environment. This method dramatically expands the range of bacterial species amenable to laboratory study and potential antibiotic production. Teixobactin’s mechanism of action is particularly noteworthy; it binds with lipid II, a key building block in the formation of bacterial cell walls. This interaction inhibits the cell wall assembly, leading to the death of the bacteria. This antibiotic has demonstrated potent activity against a variety of Gram-positive pathogens, including MRSA and VRE. The targeting of a highly conserved component in bacterial cell wall synthesis by teixobactin is a strategic advantage, as it minimizes the bacterial ability to develop resistance [57]. If teixobactin demonstrates a safer renal profile compared to vancomycin, it could become an excellent option for elderly patients, who are particularly vulnerable to acute kidney injury, including a broader coverage for vancomycin-resistant organisms [58].

Cefiderocol represents a significant advancement in the treatment of infections caused by Gram-negative bacteria, particularly those resistant to carbapenems. This innovative antibiotic is classified as a siderophore cephalosporin, and it employs a unique mechanism of action that capitalizes on the bacteria’s own iron transport mechanisms. By binding to free iron in the environment, cefiderocol is then actively transported into bacterial cells through their iron uptake channels, which are typically used by bacteria to acquire this essential nutrient. This Trojan horse strategy allows cefiderocol to circumvent common bacterial resistance mechanisms, such as mutations that reduce drug uptake through porin channels or increase drug efflux. Its efficacy has been demonstrated against a broad spectrum of difficult-to-treat pathogens, including CRE and *P. aeruginosa* [59]. Based on clinical trials, cefiderocol is indicated for the management of complicated UTI, hospital-acquired pneumonia, and ventilator-associated pneumonia. The incidence of these infections increases with age, and specifically, females aged 65+ have a double risk for UTI compared to younger females [60]. Coupled with its relatively safe profile, cefiderocol is a good option for MDRO-related infections in geriatric patients.

*A. baumannii*, recognized as a critical threat by global health organizations, poses a formidable challenge due to its high resistance to carbapenem antibiotics, making infections difficult to treat and control, particularly in hospital settings. Advancing age is a significant risk factor for *A. baumannii* infections, with the elderly accounting for approximately 70% of cases and experiencing a worse prognosis compared to younger individuals [61]. Given the high rates of antibiotic resistance exhibited by *A. baumannii*, particularly to carbapenems and other broad-spectrum agents, these infections are extremely difficult to treat. This creates an urgent need for new therapeutic options. Murepavadin, a novel macrocyclic peptide antibiotic, targets the lipopolysaccharide transporter in *A. baumannii*, a mechanism vital for bacterial cell wall synthesis. This targeted approach disrupts the outer membrane assembly, presenting a promising strategy against carbapenem-resistant strains. Such specificity not only addresses resistance mechanisms directly but also offers potential advantages in preserving host microbiota [62].

Meropenem-vaborbactam is a potent combination that pairs meropenem, a broad-spectrum carbapenem antibiotic known for its efficacy against Gram-negative bacteria, with vaborbactam, a beta-lactamase inhibitor that specifically targets the KPC. By inhibiting KPC, vaborbactam protects meropenem from enzymatic degradation, thereby preserving its antibacterial activity. This synergistic combination enhances the effectiveness of meropenem against a wide range of carbapenem-resistant organisms, including CRE [63]. This has especially important implications for the management of CRE infections in geriatric patients, as they are particularly prevalent among long-term care facility residents compared to those in the community [64].

Plazomicin is a novel aminoglycoside designed to combat infections caused by MDROs, particularly CRE. Its structure has been modified to evade aminoglycoside-modifying enzymes, which render older aminoglycosides like gentamicin and tobramycin ineffective. In addition to its efficacy against *Enterobacteriaceae*, plazomicin has demonstrated activity comparable to amikacin against *P. aeruginosa* and *A. baumannii*, though its activity against the latter has been more variable. Against *Staphylococcus* spp., plazomicin outperformed amikacin but was less effective than gentamicin and tobramycin. In terms of safety, although aminoglycosides are typically associated with nephrotoxicity, plazomicin exhibited a lower incidence of renal toxicity in clinical trials [65]. The increased prevalence of CRE infections in the elderly, coupled with the relatively lower risk of nephrotoxicity associated with plazomicin, makes it a promising choice for managing these infections in this vulnerable population [64].

Ceftolozane/tazobactam is a combination of a fifth-generation cephalosporin and a beta-lactamase inhibitor, designed to combat a broad spectrum of Gram-negative bacteria. It has demonstrated remarkable efficacy, particularly in treating infections caused by *P. aeruginosa*, including MDR and XDR strains, where it often outperforms other beta-lactams. In the case of *Enterobacteriaceae*, ceftolozane/tazobactam is especially effective against ESBL-producing organisms, offering a crucial alternative to carbapenems. However, it is not effective against CRE [66].

Ceftazidime/avibactam is a powerful antibiotic combination targeting MDR Gram-negative organisms, including *Enterobacteriaceae* and *P. aeruginosa*. The inclusion of avibactam, a novel β-lactamase inhibitor, broadens the spectrum of ceftazidime’s activity by protecting it from degradation by a range of β-lactamases, including ESBL, AmpC, KPC, and OXA-48 enzymes. However, ceftazidime/avibactam lacks activity against strains producing MBLs such as NDM, which remain resistant to this combination. In terms of tolerability, clinical trials have demonstrated that ceftazidime/avibactam is generally well-tolerated, with a safety profile consistent with ceftazidime alone [67].

It is noteworthy that the elderly account for over 50% of infections caused by ESBL-producing organisms [68]. As a result, antibiotics like ceftolozane/tazobactam and ceftazidime/avibactam, which have a relatively safe profile, play a crucial role in managing these infections in geriatric patients.

A summary of the novel antibiotics for MDRO is presented in Table 3.

### 4.2. Bacteriophage Therapy

Recent advancements in phage therapy offer promising alternatives to traditional antibiotics, particularly for managing infections from MDROs, which are of growing concern in the elderly population. Phage therapy utilizes bacteriophages, viruses that specifically infect bacteria, to target and lyse antibiotic-resistant pathogens, providing a highly targeted approach to infection control. This precision is especially beneficial for elderly patients, who are more vulnerable to infections from MDROs and often have a higher risk of adverse effects from broad-spectrum antibiotics [69]. Furthermore, phage therapy, by preserving the host microbiota and minimizing off-target effects, could reduce complications such as CDI, which predominantly affect elderly patients who account for over 70–80% of cases [70]. Additionally, the ability of phages to evolve alongside bacteria presents a dynamic method to counteract the rapid emergence of resistance. However, challenges such as phage–host specificity, the potential for immune responses to phages, and regulatory hurdles remain. Despite these challenges, phage therapy represents a viable and innovative approach to combat the growing threat of antibiotic resistance and provide potentially safe and effective therapeutic for the particularly vulnerable elderly population [71]. Future clinical trials should focus on the efficacy and safety of phage therapy, especially in elderly patients with MDROs.

### 4.3. Antivirulence Therapies

Antivirulence therapies offer a promising alternative to traditional antibiotics by targeting bacterial virulence factors—molecules that contribute to the pathogen’s ability to cause disease—rather than directly killing the bacteria. By disarming pathogens instead of killing them, antivirulence therapies reduce the selective pressure for resistance to develop.

One of the primary targets for antivirulence therapies is quorum sensing, a bacterial communication system that regulates the expression of virulence factors and the formation of biofilms. Quorum sensing inhibitors disrupt bacterial communication, preventing the coordination of virulence factor production and biofilm formation. This approach has shown promise in treating infections caused by *P. aeruginosa*, where quorum sensing plays a critical role in biofilm development and chronic infection. Furanones and acyl-homoserine lactone analogs have been identified as potential quorum sensing inhibitors and have been shown to reduce biofilm formation and enhance the efficacy of antibiotics against biofilm-associated infections [72]. The potential use of quorum sensing inhibitors against *P. aeruginosa* infections is particularly important for elderly patients with recent healthcare contact, as they are frequently affected by this pathogen [73]. Notably, a retrospective study of elderly patients with community-acquired pneumonia and positive sputum cultures for *P. aeruginosa* found that coverage with antipseudomonal antibiotics did not significantly reduce mortality [74]. In this context, novel approaches such as quorum sensing inhibitors could improve outcomes in elderly patients with *P. aeruginosa* infections and should be a focus of future clinical trials.

Another antivirulence strategy involves the use of toxin inhibitors. Bezlotoxumab is a prime example of this strategy, targeting the toxin B produced by *C. difficile*. This monoclonal antibody binds to the toxin, preventing it from interacting with colonic epithelial cells and causing inflammation and diarrhea. The efficacy of bezlotoxumab was demonstrated in a pivotal phase III trial, which indicated a significant reduction in the recurrence of CDI among high-risk groups, particularly elderly patients who are often more susceptible to complications and recurrent infections. The trial also highlighted the potential of bezlotoxumab as a supplementary treatment alongside standard antibiotic regimens, suggesting that such a targeted antivirulence approach could reduce the reliance on antibiotics and help mitigate the risk of developing further antibiotic resistance [75]. This approach is particularly vital for recurrent CDI, with very important implications for geriatric patients, who account for 70–80% of CDI infections and 90% of CDI-related mortality [70,76].

Additionally, antitoxin antibodies are being developed for the prevention of infections caused by resistant bacteria. AR-301 is a fully human IgG1 monoclonal antibody targeting the alpha-toxin of *S. aureus* that was safe and tolerable in a phase 2 trial of patients with severe pneumonia, while the results of phase 3 trials are pending [77]. AR-501, an inhaled gallium citrate with broad-spectrum anti-infective properties, is being tested in phase 2a for treating *P. aeruginosa* infections in cystic fibrosis patients [78]. AR-401 focuses on combating Gram-negative *A. baumannii* infections and is in the preclinical stage [79]. Lastly, AR-101, a human IgM mAb, is in phase 2, targeting a specific liposaccharide serotype of *P. aeruginosa* commonly associated with HAP [78].

### 4.4. Probiotics and Fecal Microbiota Transplantation

Dysbiosis refers to alterations in the composition and function of the microbiome. The gut microbiome, composed of trillions of microorganisms, is essential for regulating metabolism, immunity, and overall health. Dysbiosis has been associated with aging, chronic conditions, and antibiotic use and can lead to a variety of age-related diseases. Furthermore, dysbiosis is often linked with colonization by MDROs, especially in elderly patients with healthcare contact or nursing home residency [80].

Probiotics are live microorganisms that can confer health benefits to the host, particularly by restoring the balance of the gut microbiome, potentially eliminating pathogenic and resistant bacterial strains. Lactobacillus and Bifidobacterium strains are among the most commonly used probiotics, and several studies have shown that they can reduce the incidence of antibiotic-associated diarrhea and CDI in elderly patients. A meta-analysis examined the role of probiotics in both the primary and secondary prevention of CDI through a systematic review and meta-analysis involving 25 trials. This study revealed that probiotics like *Saccharomyces boulardii* and various Lactobacillus species can significantly reduce the incidence of CDI in primary prevention. However, these probiotics did not demonstrate a notable effect in secondary prevention scenarios [81]. Furthermore, an integrative review assessing the efficacy of probiotics in preventing CDI in older adults demonstrated a reduction in CDI incidence with probiotic use, specifically *S. boulardii* [82]. One study reported a decrease in CDI rates from 3.6% to 1.5% in one hospital where patients received *S. boulardii* alongside antibiotics [83]. Another study found that *S. boulardii* reduced hospital-onset CDI (HO-CDI) risk, particularly when administered within 24 h of starting antibiotics [84]. Overall, while some evidence suggests probiotics, particularly *S. boulardii*, may reduce CDI risk in older adults, larger and more standardized studies are needed to confirm these findings.

Fecal microbiota transplantation (FMT) is another promising intervention for the prevention and treatment of recurrent CDI. FMT involves the transfer of fecal matter from a healthy donor to a patient with recurrent CDI, with the goal of restoring a healthy microbiome and preventing further infections. According to a recently published meta-analysis of 45 studies assessing the clinical use of FMT, the mean age of patients was 65.7 years with an average of 3.7 episodes of CDI. This study concluded that FMT demonstrated efficacy rates as high as 90% in resolving recurrent CDI in this predominantly elderly population, making it an effective alternative to prolonged antibiotic therapy [85].

### 4.5. Vaccine Development

Vaccine development is a powerful tool for both primary and secondary prevention of infections caused by MDROs and has the potential to significantly reduce hospitalization rates and mortality in the elderly population. However, the aging immune system, characterized by immunosenescence, presents major hurdles. Immunosenescence affects both innate and adaptive immunity, leading to diminished production and functionality of T cells, reduced B-cell diversity, and impaired antigen presentation. This significantly weakens the elderly’s ability to mount strong and lasting responses to traditional vaccines, making them more vulnerable to infections, even with immunization [86].

In addition to these immunological challenges, developing vaccines for MDROs is particularly complex due to the highly adaptive nature of these pathogens. Many MDROs, such as *K. pneumoniae*, *A. baumannii*, and *P. aeruginosa*, possess sophisticated mechanisms like biofilm formation, capsular polysaccharide variation, and immune evasion strategies that make it difficult for vaccines to effectively target and neutralize them. Moreover, the diversity in bacterial strains and rapid evolution of resistance factors require the development of vaccines that can address not only a single strain but offer broad protection against various serotypes or variants [20,21,22,23,28,29,30,31,32,33,34].

To overcome these obstacles, innovative approaches, including the use of novel adjuvants, multi-epitope vaccines, and ML-driven antigen discovery, are essential.

#### 4.5.1. Multi-Epitope Vaccines Using Immuno-informatics and mRNA Technologies

Multi-epitope vaccines have gained attention in combating pathogens like *P. aeruginosa* and *A. baumannii* due to their ability to target multiple antigenic components, increasing immune response breadth.

For *A. baumannii*, a major MDRO causing life-threatening infections in hospital settings, vaccine development is challenging but essential, especially for the elderly. Recent advances utilize immuno-informatics to design vaccines incorporating B-cell, cytotoxic T-cell, and helper T-cell epitopes from conserved lipopolysaccharide assembly proteins like LptE and LptD, which are vital for bacterial membrane stability. These epitopes trigger robust humoral and cellular immune responses, making them ideal for broader protection [87]. To boost the effectiveness of these multi-epitope vaccines, adjuvants like human beta-defensin 3 are employed to enhance the immune response in older adults. This approach ensures that vaccines can overcome immunosenescence, helping elicit strong primary and memory immune responses. The ability of multi-epitope vaccines to provide broad protection, particularly in hospital environments, makes them a crucial development in the fight against *A. baumannii* and other MDROs that affect vulnerable populations like the elderly [87].

For *P. aeruginosa*, a novel multi-epitope mRNA vaccine was designed using immuno-informatics to target key surface proteins involved in bacterial adhesion and immune evasion, such as OprF, OprI, and FliC. This mRNA vaccine aims to elicit strong humoral and cellular immune responses by delivering selected antigenic epitopes that can activate B-cell, T-helper cell, and cytotoxic T-cell pathways. In in silico studies, this vaccine construct demonstrated high antigenicity, non-allergenicity, and stability, providing a potentially scalable and cost-effective solution against *P. aeruginosa* infections [88]. Additionally, molecular docking and immune simulation studies revealed that this mRNA vaccine effectively binds to TLR4, essential for triggering innate and adaptive immunity, further enhancing its efficacy. Immune simulations predicted strong IgM and IgG responses, indicating robust memory formation. While these findings are promising, the proposed vaccine requires further in vitro and in vivo validation to confirm its safety and effectiveness against *P. aeruginosa* in real-world settings [88].

There is currently no clinical evidence supporting the efficacy of multi-epitope vaccines specifically in the elderly population. However, these vaccines offer potential benefits for addressing immunosenescence. Multi-epitope vaccines can address this challenge by incorporating multiple epitopes that activate both T-cell and B-cell responses [88,89]. This approach potentially leads to more robust immunity, as these vaccines engage various components of the immune system, which may compensate for age-related deficits. Furthermore, multi-epitope vaccines can be customized to target conserved regions of pathogens that are critical for their survival and less susceptible to mutations. These conserved epitopes can elicit effective immune responses, even in elderly immunosenescent patients [88,89]. Thus, multi-epitope vaccines may offer a promising avenue for enhancing protection against infections in older adults by overcoming limitations seen with conventional vaccines. Future studies focusing on clinical trials in elderly populations will be crucial to establish the efficacy and safety of these vaccines in this high-risk group.

#### 4.5.2. Nanoparticle-Based Vaccines

Nanoparticle-based vaccines have emerged as a promising alternative to combat bacterial infections from MDROs, addressing the limitations of traditional vaccines and antibiotics. These vaccines utilize nanomaterials such as polymeric nanoparticles, gold nanoparticles, and bacterial outer membrane vesicles to deliver antigens effectively while enhancing immune responses. Nanoparticles offer advantages like high stability, controlled antigen release, and targeted immune stimulation, improving both humoral and cellular immunity. Moreover, they reduce the need for traditional adjuvants by serving as both carriers and immune stimulators, which can minimize side effects and enhance vaccine efficacy. This innovation offers a significant step forward in addressing the global threat of antimicrobial resistance [90,91].

By incorporating these nanoparticles, vaccines can better mimic pathogens, prolonging immune activation and ensuring a more robust defense. Notably, gold nanoparticles (AuNPs) and polymeric systems, like PLGA, have demonstrated the ability to enhance the production of cytokines and antibody responses, providing long-lasting protection against pathogens. These nanoparticle vaccines also allow for flexibility in administration routes and improve thermostability, making them ideal for global distribution [90,91].

For example, chitosan nanoparticles have been employed to develop vaccines targeting *E. coli*, including drug-resistant strains like enterohemorrhagic *E. coli* (EHEC). In a study, chitosan nanoparticles encapsulating recombinant antigens from *E. coli* successfully induced both humoral and mucosal immune responses in mice, leading to reduced infections caused by EHEC [92]. Additionally, gold nanoparticles have been investigated for their potential to enhance the immune response against *S. aureus*, particularly MRSA, which is a major cause of nosocomial infections. These nanoparticles can improve vaccine efficacy by targeting specific virulence factors such as pore-forming toxins, providing a novel platform for addressing MDROs like MRSA [91].

Although there is no current clinical evidence, nanoparticle-based vaccines offer several potential benefits for elderly populations, addressing key challenges posed by immunosenescence. Based on preclinical evidence, these vaccines can enhance immunogenicity by efficiently delivering antigens to immune cells, particularly dendritic cells, which are crucial for initiating strong immune responses. Furthermore, nanoparticles can act as adjuvants, boosting the body’s response to the vaccine, which is particularly useful in older adults who often have weaker responses to conventional vaccines [93]. These advantages make nanoparticle-based vaccines a promising tool for improving vaccine efficacy and protecting elderly individuals against infections from MDROs.

#### 4.5.3. Reverse Vaccinology and Antigen Discovery

Reverse vaccinology is a novel approach that leverages genomic, proteomic, and immunomic technologies to expedite vaccine development, particularly for MDROs. Traditional vaccine development processes, which often rely on animal models and phase 3 clinical trials for efficacy evaluation, face significant challenges due to the unreliability of animal models and the absence of clear immunological correlates of protection. Reverse vaccinology overcomes these obstacles by identifying potential vaccine antigens through bioinformatics and by evaluating vaccine efficacy earlier in clinical development, in populations with a high incidence of disease [94]. This method allows for the identification of immune correlates of protection earlier in the vaccine development process, facilitating faster refinement of vaccine dosages, schedules, and formulations. By doing so, reverse vaccine development reduces the risk of large-scale trial failures and accelerates the delivery of life-saving vaccines for MDROs such as *S. aureus* and *P. aeruginosa* [94].

For *P. aeruginosa*, reverse vaccinology has identified key proteins such as the Type III secretion system proteins, which are crucial for its pathogenicity. By targeting these proteins, potential vaccines could disrupt the bacterium’s ability to inject toxins into host cells, effectively neutralizing one of its primary mechanisms of infection. These vaccine candidates might also include other virulence factors like alginate and pilin, which are involved in biofilm formation and adhesion, respectively, addressing both acute and chronic infections by targeting the bacterial structures critical for colonization and persistence [95].

For *S. aureus*, reverse vaccinology focuses on surface proteins like ClfA and IsdB, which mediate adhesion to host tissues, and the iron-regulated surface determinant proteins that help the bacterium acquire essential nutrients. Targeting these molecules can prevent colonization and dissemination within the host. Additionally, toxins such as alpha-toxin are considered for inclusion in vaccines to neutralize their cytotoxic effects. This approach aims to disarm the bacteria, limiting their ability to evade the immune system and cause severe infections, particularly in hospital settings where MRSA is a significant concern [95].

#### 4.5.4. Lipopeptide Adjuvants

The development of vaccines for MDROs necessitates innovative approaches to enhance immunogenicity and efficacy, and lipopeptides show promise as vaccine adjuvants in this area. Their ability to act as immune modulators by activating pattern recognition receptors on antigen-presenting cells makes them particularly useful. For instance, Pam3CSK4 and Pam2CSK4 are synthetic lipopeptides that have been utilized to activate TLR2/TLR1 and TLR2/TLR6, respectively, showing promise in vaccine formulations against *P. aeruginosa* [92]. These lipopeptides have been included in vaccine studies to provoke a balanced Th1/Th17 immune response, which is crucial for defending against bacterial infections. Additionally, the use of lipopeptides in combination with other adjuvants like CpG can synergistically enhance the immunogenicity of vaccines, providing a stronger and more durable immune response against MDROs, such as *A. baumannii* and *K. pneumoniae* [96]. These strategies are especially important for immunosenescent elderly, in whom strategies for enhanced T-cell activation is crucial for an effective immune response following vaccination.

#### 4.5.5. Other Strategies to Enhance Immunity Following Vaccination

To boost vaccine efficacy in the elderly, several additional strategies are being explored as summarized by a recently published comprehensive review. Increasing antigen doses in vaccines has proven effective in enhancing immune responses. Multivalent vaccines that cover multiple strains or antigens offer broader protection, which is particularly important for age-related immune decline. The use of adjuvants like MF59 and AS03, which stimulate stronger immune responses by enhancing antigen presentation and activating immune cells, has shown promise in older populations. Additionally, inhibiting chronic inflammation through immunomodulators like metformin, which targets age-related inflammation, can improve vaccine efficacy. Addressing immunosenescence, such as by reversing T-cell aging or enhancing NK cell function, is also a key area of focus to improve immune protection in the elderly. These approaches aim to overcome the challenges posed by immunosenescence and offer more robust and long-lasting immune responses [86].

### 4.6. Antimicrobial Stewardship Programs

Antibiotic stewardship has gained increasing attention as an essential component of healthcare, particularly for addressing the growing issue of infections from MDROs. Antimicrobial stewardship programs (ASPs) aim to reduce the misuse of antibiotics by ensuring that patients receive the right drug, dose, and duration based on current guidelines, which in turn helps reduce the development of resistance and improves patient outcomes [97,98,99].

One of the main challenges in antibiotic stewardship, particularly for older adults, is the complexity of diagnosing infections in this population. Older adults often present with atypical symptoms, which can lead to diagnostic uncertainty and the unnecessary use of broad-spectrum antibiotics as a precautionary measure [98]. In the ambulatory care setting, where the majority of antibiotic prescribing occurs, older adults have the highest prescription rates, with studies showing that over 46% of antibiotics prescribed for non-bacterial respiratory infections are unnecessary. Factors like time pressures, variability in provider practice, and a “better safe than sorry” mindset can drive inappropriate antibiotic use. This is compounded by the fact that older adults are at greater risk of adverse drug events due to polypharmacy and age-related physiological changes, such as impaired renal function, which can increase toxicity and drug interactions [98].

The implementation of ASPs has shown significant promise in reducing these issues, particularly in the hospitalized elderly population. A study focusing on rigorous ASP interventions demonstrated a reduction in 30-day hospital readmission rates from 24.9% to 9.3% and a decrease in CDI-related readmissions from 2.4% to 0.3% [99]. This was achieved through interventions such as de-escalating empirical therapy, adjusting the duration of antibiotic treatment, and daily review of microbiological data to ensure appropriate prescribing. Additionally, mortality rates among elderly patients who received ASP interventions decreased from 9.6% to 5.4%, underscoring the safety and effectiveness of these programs. Not only did these ASPs improve clinical outcomes, but they also resulted in substantial cost savings, with antibiotic expenditure per patient day dropping from USD 23.33 to USD 4.37 in a six-month period [99].

Several strategies can be employed to enhance antibiotic stewardship across different care settings. In ambulatory clinics, interventions like clinician education, audit, and feedback and clinical decision-support systems have been effective in improving prescribing behaviors [98]. For example, clinician education programs that incorporate active learning and public commitments to stewardship have shown promise in reducing unnecessary antibiotic use [98]. In hospital settings, daily ASP reviews conducted by interdisciplinary teams, including pharmacists and infectious disease specialists, have been particularly successful in optimizing antibiotic use, especially for complex conditions like pneumonia and UTI [100]. Ultimately, the success of these strategies relies on a comprehensive approach, combining clinical guidelines, personalized patient care, and system-level changes to improve antibiotic prescribing practices and combat the growing threat of infections from MDROs [97,98,99].

### 4.7. Advanced Diagnostic Techniques

Rapid and accurate diagnostics are essential for guiding appropriate antibiotic use and preventing the overuse of broad-spectrum antibiotics. Recent advances in diagnostic technologies are providing clinicians with the tools to rapidly detect resistant organisms and tailor antibiotic therapy to the specific pathogen.

Nucleic acid amplification tests (NAATs) provide a rapid and effective method for diagnosing MDRO bacteria such as MRSA and KPC *Enterobacteriaceae*. The use of NAATs such as real-time PCR has significantly reduced the time required to detect and confirm the presence of resistance genes, facilitating quicker clinical decisions [100,101]. For example, the detection of KPC, NDM, OXA-48, and imipenemase-type (IMP-type MBL) carbapenemases in *Enterobacteriaceae* can be rapidly achieved using recombinase polymerase amplification combined with lateral flow strip, which provides results within 30 min without the need for specialized equipment. This method not only identifies the carbapenemase genes with high specificity and sensitivity but also allows for the immediate implementation of appropriate antimicrobial therapy and infection-control measures [100]. Similarly, rapid detection technologies for MRSA leverage NAATs to ascertain the presence of the *mecA* gene, thereby enabling timely and effective treatment interventions [101].

Another innovative diagnostic approach is next-generation sequencing (NGS), which can rapidly analyze the entire genome of a bacterial pathogen, providing comprehensive information about its resistance profile. This strategy has significant applications in diagnosing MDR-TB, as evidenced by a systematic review and meta-analysis that evaluated the effectiveness of targeted NGS compared to typical susceptibility tests. This study demonstrated that targeted NGS achieved an impressive sensitivity of 99.1% for rifampin and robust sensitivities of 93.1% for ethambutol and 99.4% for amikacin. Targeted NGS facilitates direct testing on clinical samples, offering a substantial advantage in rapid diagnosis crucial for effective MDR TB management [102]. Despite its efficacy, the adoption of targeted NGS faces challenges, particularly in regions burdened heavily by TB, where access to such advanced technologies remains limited.

Finally, matrix-assisted laser desorption/ionization–time of flight mass spectrometry (MALDI-TOF MS) has emerged as a revolutionary tool in clinical microbiology for the rapid identification of MDROs. This technology offers significant advantages over traditional methods, such as speed, accuracy, and cost effectiveness. MALDI-TOF MS can identify pathogenic microorganisms directly from clinical samples like blood, urine, and cerebrospinal fluid, enhancing the speed of diagnostic processes crucial for effective treatment [103]. For instance, MALDI-TOF MS has been instrumental in the rapid identification of MDROs such as MRSA by detecting specific protein markers indicative of resistance. Similarly, it facilitated the detection of carbapenem-resistant *A. baumannii* by identifying unique protein peaks associated with resistance mechanisms. The technology has also proven effective in distinguishing between different strains of *M. tuberculosis*, which is vital for tailoring appropriate treatment strategies [103]. Overall, MALDI-TOF MS stands out for its ability to quickly and accurately detect MDROs, thereby playing a crucial role in managing infectious diseases effectively, reducing treatment delays, and improving patient outcomes.

There is lack of evidence directly focusing on the use of advanced diagnostic techniques such as NAATs, NGS, or MALDI-TOF MS specifically for the elderly, but these technologies offer promising benefits for managing infections in this population. In emergency settings, these techniques can rapidly detect MDROs, helping avoid the misuse of broad-spectrum antibiotics when they are not necessary, which is particularly important for elderly patients who often present with atypical disease symptoms. Additionally, these diagnostics can quickly screen for resistant pathogens like MRSA in nursing home residents, facilitating the cohorting of patients with similar pathogens and preventing cross-transmission of MDROs [104]. Future studies should explore these applications in the elderly, offering potential for more targeted interventions and improved infection control in this vulnerable population.

A summary of the strategies to combat antibiotic resistance in the elderly are summarized in Table 4.

## 5. The Role of ML in Combating Antibiotic Resistance in the Elderly

ML is emerging as a powerful tool in the fight against antibiotic resistance, particularly in elderly populations. ML algorithms can analyze vast amounts of clinical data, identify patterns, and make predictions that improve the diagnosis, treatment, and prevention of antibiotic-resistant infections. Below, the role of ML in combating antibiotic resistance is explored in the context of specific clinical applications.

### 5.1. ML in Diagnosis

ML algorithms can aid in the differential diagnosis of bacterial from viral pneumonia, aiming to avoid antibiotic misuse. According to a recent study, a novel diagnostic model has been proposed that that synthesizes convolutional neural networks (CNN) with extreme gradient boosting (XGboost) to effectively discern between bacterial and viral forms of pneumonia through chest X-ray imagery [105]. This method leverages the CNN’s capacity for intricate feature extraction from the images, which captures the distinct radiographic signatures that differentiate bacterial from viral pneumonia. Following feature extraction, the XGboost algorithm classifies these features with a high degree of precision, facilitating an accurate diagnosis. Remarkably, this model achieves an accuracy of 87% and processes each image within a mere 7 s, thereby demonstrating its utility in fast-paced clinical environments. The combined specificity of 89% and sensitivity of 85% achieved by the model markedly enhance diagnostic reliability, reducing the dependence on subjective radiological interpretation and supporting rapid clinical decision making [105].

As already stated, infections in the elderly often manifest atypically, and symptoms that appear to be infectious can also be caused by non-infectious precipitants such as medication side effects. In this context, hybrid diagnostic models that combine ML techniques like CNNs and support vector machines (SVM) can be instrumental. These models analyze a variety of clinical inputs, including medical histories, lab results, and imaging data, enabling them to discern subtle distinctions between infectious diseases and symptoms stemming from other causes [106]. Utilizing such advanced diagnostic tools can also significantly contribute to reducing antibiotic overuse and misuse both in community settings and hospitals, thus helping to mitigate the risk of antibiotic resistance and ensuring that antibiotics are reserved for cases where they are truly needed.

### 5.2. Predictive Models for Antibiotic Resistance and Antibiotic Stewardship

Recent advancements in ML have significantly bolstered antimicrobial stewardship programs (ASPs), enhancing their capability to manage antibiotic prescriptions and combat antimicrobial resistance. The integration of ML models in ASPs enables the prediction of antibiotic resistance patterns and optimizes antibiotic therapy, especially in complex cases where traditional methods may falter due to delayed susceptibility results. ML models can analyze a wide range of variables, such as patient demographics, infection site, previous treatment history, and microbial characteristics, to generate real-time predictions about resistance. For example, algorithms like random forests and support vector machines have been shown to predict antimicrobial resistance with high accuracy, allowing clinicians to initiate more targeted therapies earlier, potentially reducing the use of broad-spectrum antibiotics and improving patient outcomes [107]. Additionally, ML models can monitor antimicrobial usage patterns across different settings and identify inappropriate prescriptions, as seen in studies where ML accurately flagged misuse of antibiotics like piperacillin-tazobactam with precision rates as high as 74% [108].

Beyond prediction, ML also assists in personalized treatment strategies by dynamically adjusting antibiotic choices based on real-time data input, such as ongoing patient responses or newly emerging resistance trends. These capabilities are particularly valuable in high-risk environments like ICUs, where rapid decision making is crucial. In fact, ML models have been employed to assess treatment outcomes under different scenarios (e.g., stopping or continuing antibiotic therapy), providing data-driven recommendations that help balance effectiveness with minimizing unnecessary antibiotic exposure [107]. The use of explainable AI methods, such as SHAP (Shapley Additive exPlanations), further improves clinical adoption by clarifying the reasoning behind model predictions, thus helping stewardship teams understand and trust the model’s outputs [108]. Moreover, ML’s ability to handle vast, multidimensional datasets allows ASPs to quickly identify patterns and trends in antibiotic resistance, something traditional methods might overlook. This capability facilitates more precise interventions in both inpatient and outpatient settings, improving the overall quality of care while actively reducing the development of antimicrobial resistance. By integrating ML into ASPs, healthcare systems can not only improve prescription accuracy but also contribute to the global fight against antimicrobial resistance by promoting responsible antibiotic use [107].

Traditional susceptibility tests can delay the initiation of effective treatment, often leading to reliance on empirical antibiotic therapy that may not be ideal. ML presents a potential solution by predicting resistance patterns, facilitating earlier and more precise therapeutic interventions. Recent applications of ML in ICU settings include the use of algorithms such as support vector machines, random forests, and multilayer perceptrons. These algorithms utilize basic data elements like Gram stains, sample types, and patient demographics to predict antibiotic resistance [109,110,111]. Achieving accuracies as high as 72.6%, these models provide valuable insights ahead of traditional lab results. Moreover, platforms like Microsoft Azure’s AutoML have demonstrated effectiveness in streamlining the model selection process. For instance, an ensemble model trained on 11,496 data points achieved an impressive area under the curve score of 0.850 by employing the synthetic minority oversampling technique to address data imbalances. Such ML capabilities can guide clinicians in choosing more accurate empirical therapies, especially for managing pathogens like *K. pneumoniae* and *P. aeruginosa*, thereby reducing the use of unnecessary broad-spectrum antibiotics. As ML technologies advance, incorporating patient-specific clinical and genetic data could further refine their predictive accuracy, offering more tailored and effective treatment strategies for elderly patients prone to severe infection-related outcomes [109,110,111].

### 5.3. ML in Vaccine Development

ML plays a pivotal role in accelerating vaccine development by enabling the rapid identification of antigen candidates and predicting immune responses. Integration of multi-omics technologies with ML algorithms is a promising strategy than can provide useful solutions in research and personalized medicine [80]. For example, ML algorithms can process large datasets derived from genomic and proteomic studies to identify microbial components that trigger immune responses, as seen in the use of reverse vaccinology techniques. Reverse vaccinology, bolstered by ML, has demonstrated the potential to fast-track vaccine design by screening pathogen genomes without the need for traditional culture methods, reducing both time and cost in vaccine research [112]. Specifically, ML-driven tools like VaxiJen and Vaxign-ML have been instrumental in predicting protein antigens that can elicit strong immune responses by analyzing physicochemical properties and genomic sequences of pathogens [113]. Moreover, during the COVID-19 pandemic, ML approaches were essential in rapidly developing mRNA vaccines by analyzing viral mutations and optimizing vaccine efficacy. These advancements showcase how ML not only expedites the discovery of vaccines but also enhances their accuracy and effectiveness, particularly against MDROs [112,113].

### 5.4. ML in Antibiotic Development

ML is revolutionizing antibiotic development, particularly in the fight against MDROs. By using ML models, researchers can efficiently explore vast chemical spaces to identify potential antibiotic compounds that might be missed through conventional methods. For instance, ML was instrumental in screening approximately 7500 molecules to discover Abaucin, a novel antibiotic with targeted activity against the MDR *A. baumannii*. This discovery, enabled by neural network models, highlights the role of ML in identifying compounds that inhibit bacterial growth and can be further tested in preclinical models [114]. Moreover, explainable deep learning approaches, such as graph neural networks, have been developed to predict antibiotic activity and cytotoxicity for millions of compounds. This approach allows scientists to identify chemical substructures that are responsible for antibiotic activity, leading to the discovery of new structural classes of antibiotics. These classes are potentially capable of combating MRSA and other MDROs, providing a significant advantage in the ongoing antibiotic resistance crisis [114]. By integrating ML models that are not only predictive but also explainable, researchers gain valuable chemical insights, speeding up the discovery of effective, low-toxicity antibiotics.

## 6. Conclusions

Antibiotic resistance in the elderly is a growing healthcare crisis, mediated by factors such as immunosenescence, multimorbidity, and frequent healthcare exposure, which make this population especially vulnerable to MDROs such as MRSA, VRE, and CRE. Traditional antibiotics are becoming less effective, necessitating novel approaches such as next-generation antibiotics (e.g., teixobactin and cefiderocol), bacteriophage therapy, and antivirulence treatments. Rapid molecular diagnostics, including NAAT and NGS, enable timely detection of resistant pathogens, facilitating precise treatment. In parallel, vaccine development—through multi-epitope and nanoparticle-based vaccines—offers preventive strategies against MDROs. ASPs are crucial in optimizing antibiotic use, reducing unnecessary prescriptions, and curbing the spread of resistance. Additionally, ML enhances diagnostic accuracy, predicts resistance patterns, and aids in both personalized treatment and vaccine development.

A comprehensive approach that integrates advanced therapies, rapid diagnostics, vaccines, stewardship, and ML technologies is essential to mitigating antibiotic resistance in the elderly. These strategies, combined with ongoing research and global collaboration, will be key in addressing this urgent public health challenge.

## Figures and Tables

**Table 1:** Mechanisms of the most prevalent MDROs and their most common infections in the elderly.

MDR Bacteria	Major Mechanisms of Resistance	Common Infections	References
MRSA	Acquisition of *mecA* gene encoding altered PBP2a with low affinity for beta-lactams Efflux pumps (NorA) Biofilm formation Horizontal gene transfer	Skin, respiratory, and bloodstream infections, infective endocarditis. Particularly in hospital/long-term care settings.	[11,12,13]
VRE	*vanA*/*vanB* gene clusters modify D-Ala-D-Ala to D-Ala-D-Lac, reducing vancomycin binding Penicillinase production Biofilm formation Horizontal gene transfer	Urinary tract, bloodstream and wound infections, infective endocarditis. Particularly in hospital/long-term care settings.	[14,15,16]
*S. pneumoniae*	Alterations in PBPs Acquisition of *ermB* gene for macrolide resistance *mefA* gene encoding efflux pumps Biofilm formation	Pneumonia and meningitis.	[17,18,19]
*Enterobacteriaceae*	Production of ESBLs and carbapenemases (e.g., KPC, NDM, and OXA-48) Porin mutations (OmpK36) Efflux pumps (AcrAB-TolC) Horizontal gene transfer	Urinary tract, respiratory, bloodstream and wound infections. Particularly in hospital/long-term care settings.	[20,21,22,23]
*C. difficile*	Mutations in *gyrA* and *gyrB* genes for fluoroquinolone resistance Biofilm formation Spore formation	Healthcare-associated diarrhea/pseudomembranous colitis. Often following prolonged antibiotic use.	[24,25]
MDR/XDR *M. tuberculosis*	Mutations in *katG* (isoniazid resistance) and *rpoB* (rifampin resistance) Efflux pumps Cell wall modifications reducing drug influx	Tuberculosis. Particularly in immunocompromised or elderly patients.	[26,27]
*P. aeruginosa*	Low outer membrane permeability—porin mutations Efflux pumps (e.g., MexAB-OprM, and MexXY) Production of beta-lactamases (e.g., MBLs) Biofilm formation	Respiratory (e.g., ventilator-associated pneumonia), urinary tract, bloodstream, and wound infections. Particularly in hospital/long-term care settings.	[28,29,30]
*A. baumannii*	Beta-lactamase production (e.g., OXA) Porin mutations Biofilm formation Horizontal gene transfer of resistance genes	Respiratory (e.g., ventilator-associated pneumonia), urinary tract, bloodstream, and wound infections. Particularly in the intensive care unit.	[31,32,33,34]

Abbreviations. ESBL (extended-spectrum beta-lactamase); KPC (*Klebsiella pneumoniae* carbapenemase); MBL (metallo-beta-lactamase); MDR (multidrug-resistant); MRSA (methicillin-resistant *Staphylococcus aureus*); NDM (New Delhi metallo-beta-lactamase); OXA (oxacillinase-type carbapenemase); PBP (penicillin-binding protein); VRE (vancomycin-resistant Enterococci); XDR (extensively drug-resistant).

**Table 2:** Summary of the risk factors for MDRO-related infections in the elderly.

Risk Factors	Description	References
Immunosenescence	Gradual decline in immune function due to aging. Impaired neutrophil/macrophage activity and reduced cytokine production. Reduced T-cell production and B-cell function, limiting response to infections and vaccines. Contributes to persistence of resistant pathogens.	[35,36]
Polypharmacy	Defined as the use of 5 or more medications. Higher risk of drug interactions and antibiotic misuse. Proton pump inhibitors and other drugs cause dysbiosis, increasing susceptibility to resistant infections.	[37,38,39]
Sarcopenia and Malnutrition	Sarcopenia, the progressive, age-related loss of muscle mass, is linked to chronic inflammation and increased infection risk. Malnutrition worsens immune function and immunoglobin production. Micronutrient deficiencies (e.g., zinc and vitamin D) impair immune response.	[40,41,42]
Frailty and Decreased Mobility	Reduced physiological reserve and chronic inflammation increase the risk of severe infections. Immobility leads to pressure ulcers and hospital-acquired infections. Associated with increased use of invasive devices, which raises the risk of MDRO-related infections.	[43,44,45,46,47]
Cognitive Impairment	Cognitive dysfunction increases MDRO-related infection risk due to poor hygiene, catheter use, and difficulty following infection-control protocols. Atypical disease presentation often leads to unnecessary antibiotic use, initiating a vicious cycle of resistance.	[48,49]
Multimorbidity	Chronic conditions (e.g., diabetes mellitus, chronic kidney disease, heart failure, and chronic obstructive pulmonary disease) impair immune response and increase the risk of infections and hospitalizations, promoting colonization from MDROs. Frequent use of broad-spectrum antibiotics heightens risk of resistant infections. Immunosuppressive therapies increase susceptibility to severe infections, including those from MDROs.	[50,51,52,53,54]
Frequent Hospitalizations and LTCF Residency	Long-term care facilities and frequent hospitalizations expose elderly to MDROs. Foreign bodies (e.g., urinary catheters and central venous lines) increase MDRO-related infections. Misuse of antibiotics in healthcare settings fosters resistant organisms, creating a cycle of resistance.	[55,56]

Abbreviations. MDROs (multidrug-resistant organisms).

**Table 3:** Summary of the novel antibiotics for MDRO.

Antibiotic	Key MDRO Targets	Limitations	References
Teixobactin	MRSA VRE	Limited data on safety in elderly. Potential nephrotoxicity.	[57,58]
Cefiderocol	ESBL-producing *Enterobacteriaceae* CRE *P. aeruginosa*	Limited data in elderly. Requires cautious use in renal impairment.	[59,60]
Plazomicin	ESBL-producing *Enterobacteriaceae* CRE *P. aeruginosa*	Risk of nephrotoxicity, though lower than traditional aminoglycosides.	[65]
Ceftolozane/Tazobactam	ESBL-producing *Enterobacteriaceae* *P. aeruginosa*	Not effective against CRE.	[66]
Ceftazidime/Avibactam	ESBL-producing *Enterobacteriaceae* CRE *P. aeruginosa*	Not effective against MBLs.	[67]
Meropenem/Vaborbactam	ESBL-producing *Enterobacteriaceae* CRE *P. aeruginosa*	Caution in renal impairment; limited data in the elderly.	[63]
Murepavadin	*A. baumannii*	No clinical data for intravenous use.	[62]

Abbreviations. ESBL (extended-spectrum beta-lactamase); KPC (*Klebsiella pneumoniae* carbapenemase); MBL (metallo-beta-lactamase); MRSA (methicillin-resistant *Staphylococcus aureus*); VRE (vancomycin-resistant Enterococci).

**Table 4:** Summary of the strategies to combat antibiotic resistance in the elderly.

Strategies	Description	References
Novel antibiotics	Teixobactin targets lipid II, crucial for cell wall synthesis in Gram-positive bacteria. It offers potential for the elderly, particularly those vulnerable to acute kidney injury, by covering vancomycin-resistant organisms. Cefiderocol, a siderophore cephalosporin, enters Gram-negative bacteria via iron transport channels, acting as a Trojan horse. It is effective against CRE and *P. aeruginosa*, crucial for treating urinary tract infections and pneumonia, which are common in older adults.	[57,58,59,60,61,62,63,64,65,66,67,68]
Bacteriophage therapy	Employs bacteriophages, viruses that specifically infect and lyse bacteria, to target antibiotic-resistant pathogens. Offers a highly specific treatment preserving host microbiota and reducing the risk of complications like CDI, which disproportionately affects elderly patients (70–80% of cases). Phage therapy reduces the adverse effects of broad-spectrum antibiotics in elderly patients, who are more vulnerable to MDRO-related infections.	[69,70,71]
Antivirulence therapies	Target pathogenicity instead of bacterial survival by inhibiting virulence factors like toxins and biofilm formation. Quorum sensing inhibitors prevent bacterial communication and biofilm production, aiding in the treatment of *P. aeruginosa* infections in elderly patients, especially those with recent healthcare exposure. Bezlotoxumab neutralizes *C. difficile* toxin B, significantly reducing recurrence rates in elderly patients, who account for 90% of CDI-related mortality.	[72,73,74,75,76,77,78]
Probiotics and fecal microbiota transplantation	Probiotics like Lactobacillus and *Saccharomyces boulardii* restore the balance of gut microbiota, reducing the incidence of antibiotic-associated diarrhea and CDI in elderly patients, who often experience dysbiosis after antibiotic use. Fecal microbiota transplantation restores a healthy microbiome in elderly patients with recurrent CDI, demonstrating up to 90% efficacy in resolving infections.	[79,80,81,82,83,84]
Vaccine development	Aims to prevent infections by promoting active immunity against MDROs, crucial for the immunosenescent elderly patients. Multi-epitope and nanoparticle-based vaccines target multiple bacterial components, enhancing immune coverage and efficacy, especially against adaptive MDROs that affect vulnerable elderly populations.	[85,86,87,88,89,90,91,92,93,94,95,96]
Antimicrobial stewardship programs	It focuses on optimizing antibiotic use by ensuring the correct drug, dose, and duration, tailored to each patient. It can help reduce the misuse of antibiotics, preventing unnecessary use in elderly patients, especially given the atypical disease presentation and high rates of polypharmacy in this population.	[86,97,98]
Advanced diagnostic techniques	Modern diagnostic methods like NAATs and NGS rapidly identify pathogens and their resistance profiles, enabling more targeted antibiotic therapy and avoidance of misuse of broad-spectrum antibiotics. MALDI-TOF MS enhances rapid diagnosis of MDROs like MRSA and carbapenem-resistant *A. baumannii*.	[99,100,101,102,103]

Abbreviations. CRE (carbapenem-resistant *Enterobacteriaceae*); CDI (*Clostridioides difficile* infection); MALDI-TOF MS (matrix-assisted laser desorption/ionization–time of flight mass spectrometry); MDROs (multidrug-resistant organisms); NAATs (nucleic acid amplification tests); NGS (next-generation sequencing).

## Data Availability

Data sharing is not applicable.
